# MXene-Coated Membranes for Autonomous Solar-Driven Desalination

**DOI:** 10.1021/acsami.1c20653

**Published:** 2022-01-21

**Authors:** Mustakeem Mustakeem, Jehad K. El-Demellawi, M. Obaid, Fangwang Ming, Husam N. Alshareef, Noreddine Ghaffour

**Affiliations:** † Water Desalination and Reuse Center (WDRC), Biological and Environmental Science and Engineering Division (BESE), King Abdullah University of Science and Technology, (KAUST), Thuwal 23955-6900, Saudi Arabia; ‡ Physical Science and Engineering (PSE) Division, King Abdullah University of Science and Technology, (KAUST), Thuwal 23955-6900, Saudi Arabia

**Keywords:** membrane distillation, photothermal behavior, Ti_3_C_2_*T*_*x*,_ localized heating, 2D light-to-heat conversion material, temperature polarization, solar energy desalination

## Abstract

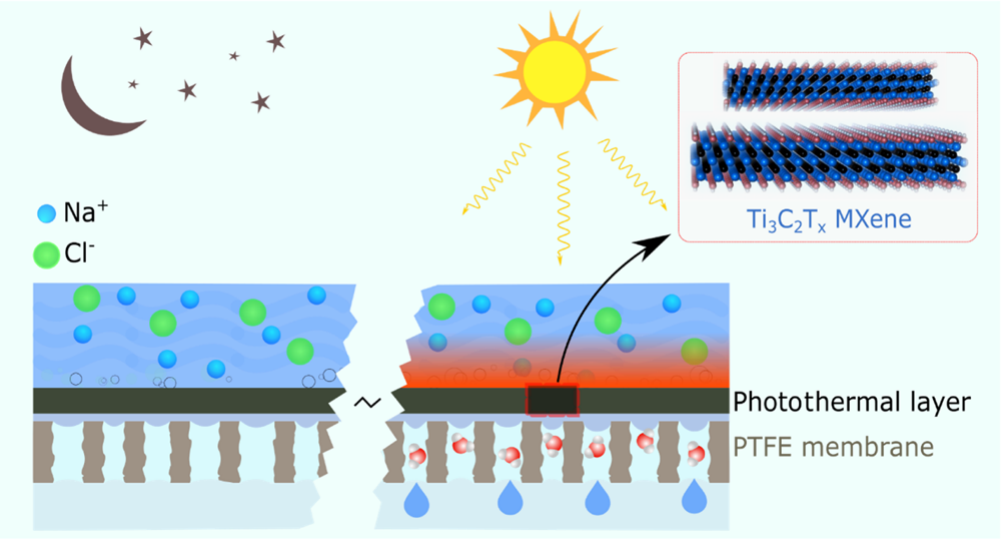

Clean water supply in off-grid locations remains a stumbling stone for socio-economic development in remote areas where solar energy is abundant. In this regard, several technologies have already introduced various solutions to the off-grid freshwater predicament; however, most of them are either costly or complex to operate. Nonetheless, photothermal membrane distillation (PMD) has emerged as a promising candidate with great potential to be autonomously driven by solar energy. Instead of using energy-intensive bulk feed heating in conventional MD systems, PMD membranes can directly harvest the incident solar light at the membrane interface as an alternative driving energy resource for the desalination process. Because of its excellent photothermal properties and stability in ionic environments, herein, Ti_3_C_2_*T*_*x*_ MXene was coated onto commercial polytetrafluoroethylene (PTFE) membranes to allow for a self-heated PMD process. An average water vapor flux of 0.77 kg/m^2^ h with an excellent temporal response under intermitting lighting and a photothermal efficiency of 65.3% were achieved by the PMD membrane under one-sun irradiation for a feed salinity of 0.36 g/L. Naturally, the efficiency of the process decreased with higher feed concentrations due to the reduction of the evaporation rate and the scattering of incident sunlight toward the membrane photothermal surface, especially at rates above 10 g/L. Notably, with such performance, 1 m^2^ of the MXene-coated PMD membrane can fulfill the recommended daily potable water intake for a household, that is, ca. 6 L/day.

## 1 Introduction

Recent reports show that water consumption has increased 600 times during the last century due to the rapid increase in global population, urban expansion, and industrialization.^1^ This increase has encouraged the water research community to find new efficient desalination and wastewater treatment technologies.^2−4^ Thus far, among the conventional technologies, mainly reverse osmosis (RO), multistage flash, and multi-effect distillation (MED), membrane distillation (MD) has been introduced as a promising technology for water desalination. A typical MD system can desalinate highly saline streams at a comparatively low temperature and pressure.^5^ Moreover, the energetic performance of an MD system can be comparable to that of existing thermal technologies.^6^ Besides, MD can be adequately integrated with other technologies, such as RO^7^ and MED,^8^ to increase water recovery. In principle, MD is driven by the difference in the vapor pressure of fluids across a hydrophobic microporous membrane. It can be categorized into several configurations that mainly differ on the permeate side.^9^ Direct contact MD (DCMD) is the most studied among these configurations due to its simplicity and comparatively high flux.^10−12^ Nevertheless, despite its advantages, the commercialization of MD technology is hindered by a few critical factors, including temperature polarization (TP), which can negatively affect the flux, rendering the process energetically inefficient.^13−17^ Furthermore, the magnitude of TP typically increases significantly with the MD module length, limiting the MD scale-up.^18,19^

In this regard, various approaches have been introduced to overcome the TP issue by either using feed spacers,^20−23^ flashed feed,^9^ or heating the feed solution locally near the membrane surface, known as localized heating.^24^ So far, the latter is the most sustainable approach, as it can be applied without affecting the feed flow hydrodynamics. Nonetheless, all the abovementioned approaches remain dependent on external heating sources. Alternatively, recent technologies have offered other routes for repressing the TP limitation by using self-heating MD membranes coated with photothermal materials.^16,25^ Generally, applying photothermal coatings on MD membrane surfaces can induce significant localized heating; a larger temperature gradient across the coated membrane is obtained.^26^ In a typical photothermal MD (PMD) process, the membrane is coated with a photothermal material that can effectively absorb solar irradiation and convert it into thermal energy. Hence, the feedwater can be heated up directly at the evaporation site, that is, the membrane–feed interface, garnering PMD systems a great potential to overcome the TP effect.^27,28^ Furthermore, using PMD membranes has demonstrated an enhancement in the vapor flux in addition to a recognizable decrease in the specific energy consumption (SEC) compared to conventional MD.^29,30^

Several photothermal materials have been tested for PMD and have exhibited high desalination performance. For example, incorporating Ag nanoparticles (NPs) into polyvinylidene fluoride (PVDF) membranes has increased the vapor flux under UV irradiation.^16,28,31^ Likewise, the deposition of Fe_3_O_4_ NPs on a PVDF-co-hexafluoropropylene nanofiber membrane exhibited a photothermal efficiency of 53% and a water flux of 0.97 kg/m^2^ h.^32^ Cao et al. (2020) used a hydroxyapatite-based nanowire membrane to distillate water under one-sun illumination. They obtained a vapor flux and a photothermal efficiency of 0.89 kg/m^2^ h and 62%, respectively.^33^ Said et al. (2019) coated carbon black NPs onto a commercial polytetrafluoroethylene (PTFE) membrane, yielding a flux value of 0.77 kg/m^2^ h.^34^ Titanium nitride (TiN)-coated PVDF membranes are another example of photothermal-induced enhanced MD performance, with a solar conversion efficiency of 64.1% and a vapor flux of 0.94 kg/m^2^ h under one-sun illumination.^35^ In addition to the previous studies involving zero- and one-dimensional (1D) photothermal materials, two-dimensional (2D) materials, such as graphene, were also considered for PMD systems. For instance, the coating of a PTFE membrane with graphene-based materials has shown a 78.6% improvement in the vapor flux under one-sun irradiation.^36^

Beyond graphene,^37^ the young family of 2D transition metal carbides/nitrides, that is, MXenes, has drawn significant interest thanks to its unique optical absorption cross section, plasmonic behavior, tunable work functions, versatile surface chemistry, excellent light-to-heat conversion efficiency, antifouling effects, and good thermal conductivity.^17,38−41^ MXenes are generally synthesized by removing the A element from their layered parent MAX phase, that is, layered ternary carbides or nitrides, where M is an early transition metal, and X is C, N, or both. They are defined by the general formula M_*n*+1_X_*n*_*T*_*x*_,^38,42^ where *T*_*x*_ represents the surface-terminated species (−F, −OH, or −O).^43,44^ Owing to their unique properties, MXene nanosheets, particularly Ti_3_C_2_*T*_*x*_, have been employed as a photothermal coating for MD membranes,^45^ benefiting from the pronounced surface plasmon (SP) oscillations at the surface of the nanosheets.^46,47^ In principle, upon the light illumination of MXene membranes at wavelengths in resonance with these SPs, the temperature gradient generated across the membrane surges due to the intrinsic SP-assisted light-to-heat conversion.^48,49^ The plasmon-induced photothermal behavior of Ti_3_C_2_*T*_*x*_ has promoted its use in PMD processes.^38,49−51^ For example, Ding et al. (2017) reported that MXene membranes exhibited 5–10 times higher water permeance during the purification of Evans blue-containing water, indicating the advantageous effect of the layered MXene nanosheets on water permeation.^52^ Although MXene-coated membranes have demonstrated high efficiencies during solar steam generation,^49,53^ the vapor flux was lower than that attained by pristine membranes due to the increased mass transfer resistance induced by the additional coating material.^26^ Recently, Tan et al. (2018) reported on the photothermal properties of MXene in a DCMD system. They coated MXene onto commercial PVDF membranes, obtaining a vapor flux and a photothermal efficiency of 10 kg/m^2^ h and 43%, respectively, under 5.8 kW/m^2^ illumination.^26^ To our knowledge, this is the only reported study using MXene as a photothermal material. However, their system simultaneously relied on both external bulk heating and photothermal-assisted self-heating under high sun irradiation (>5 times the typical solar power). Therefore, to significantly reduce the footprint of the MD process, it is highly desirable to develop MXene-based PMD systems that can solely function with a self-heating source under normal sun illumination power.

In this study, we fabricated Ti_3_C_2_*T*_*x*_-coated PTFE MD membranes with different MXene loadings. The MXene-coated MD membranes are subjected to well-controlled investigation for their performance and localized heating efficiency in a DCMD system, only driven by solar energy (one-sun irradiation). Furthermore, a detailed energy analysis, photothermal efficiency, and freshwater production efficiency are reported under different operating conditions and feedwater quality.

## 2 Materials and Methods

### 2.1 MXene Synthesis and Preparation

Suspensions of Ti_3_C_2_*T*_*x*_ MXene nanosheets were synthesized using an etching bath made of hydrofluoric acid (HF, VWR Chemicals), hydrochloric acid (HCl, Sigma-Aldrich), and deionized (DI) water (Millipore, resistivity of 18 MΩ cm) to selectively etch away the Al layer from the parent Ti_3_AlC_2_ MAX phase. In a high-density polyethylene (HDPE) bottle, 2 g of Ti_3_AlC_2_ powder (<40 μm in particle size, Carbon-Ukraine Ltd.) was added to the premade etchant solution (20 mL) and left for stirring at 40 °C for 16 h. Following the etching, the obtained suspensions of exfoliated MXene nanosheets were carefully washed in DI water through several rounds of centrifugation and decantation until a pH value of ca. 6 was attained. Delaminated MXene nanosheets were then obtained using lithium chloride (LiCl, Sigma-Aldrich) as an intercalant. Afterward, the dispersion of LiCl-intercalated Ti_3_C_2_*T*_*x*_ nanosheets was washed once with DI water, once with a DI water–methanol mixture (each 50 vol %), and then washed twice with methanol (anhydrous, 99.8%, Sigma-Aldrich) through centrifugation. Finally, the supernatant (in methanol) containing delaminated Ti_3_C_2_*T*_*x*_ nanosheets was collected and stored at ca. −15 °C for further use.

### 2.2 Fabrication of MXene-Coated Membranes

Several commercial hydrophobic PTFE microfiltration membranes (with a nominal pore size of 0.22 μm, a thickness of 200 μm, and an average contact angle of 127.7°, from Sterlitech Inc.) were coated by Ti_3_C_2_*T*_*x*_ MXene, using a conventional vacuum-assisted filtration method.^26,49,54^ Aiming for a conformal coating and an efficient attachment to the hydrophobic PTFE surface, the hydrophilic MXene nanosheets were dispersed in a less polar solvent (instead of water), that is, methanol, which has both hydrophilic and hydrophobic segments. The MXene loading per unit area was tuned using several amounts of Ti_3_C_2_*T*_*x*_ dispersions to coat the Ti_3_C_2_*T*_*x*_ nanosheets onto the PTFE membranes at different areal densities, that is, 1.4, 2.3, and 3.5 mg/cm^2^, and expressed, herein, as MX1.4, MX2.3, and MX3.5, respectively. The concentration of the used methanol dispersion of Ti_3_C_2_*T*_*x*_ nanosheets was fixed at ca. 1 mg/mL. After the deposition, the MXene-coated membranes were dried overnight under vacuum at ca. 40 °C and stored inside a vacuum desiccator for further use.

### 2.3 MXene Characterization

The morphology and elemental stoichiometry of the coated MXene films were probed using scanning electron microscopy (SEM). SEM was performed using a Zeiss Merlin workstation, equipped with an Oxford Instruments energy-dispersive X-ray (EDX) detector. Cross-sectional and top-view SEM images were taken at an electron high tension (EHT) of 5 kV and a working distance (WD) of 1.8 and 2.6 mm, respectively. For the virgin PTFE membranes, the WD was 4.7 mm. The EDX maps were acquired at an EHT of 15 kV and a WD of 8.5 mm. The quality of synthesized MXene was investigated using a combination of Raman spectroscopy and X-ray diffraction (XRD). Raman spectroscopy was conducted using a micro-Raman spectrometer (LabRAM Aramis, Horiba, Japan) equipped with green and red lasers (i.e., 532 and 633 nm, respectively) and an Olympus 50× objective lens. XRD patterns were obtained using a Bruker powder X-ray diffractometer (D8 Advance, AXS system, Germany) with Cu Kα radiation (λ = 1.5408 Å). The scanning rate was 0.02°/step (0.5 s/step) in the 2θ range of 5–50°. Ultraviolet–visible (UV–vis) spectroscopy was performed in the absorption range of 190–1000 nm using a Cary 5000 UV–vis–NIR spectrometer (Varian Inc.). A baseline correction was applied before obtaining absorption data.

### 2.4 PMD Experiments

The performance of MXene-coated membranes was tested using a custom-made acrylic MD module with an active membrane area of 0.0025 m^2^ (50 × 50 mm). The flat Ti_3_C_2_*T*_*x*_-coated membrane, housed inside the MD module, was sandwiched between the feed and coolant water flow channels. The feedwater was fed through an overhead tank at different water salinities. Meanwhile, the coolant water was recirculated (in the counter-current direction) using a gear pump (model 75211, Cole-Parmer, USA). The feedwater flow was controlled through a needle valve (Swagelok, SS-31RS4), while the coolant flow rate was fixed at 100 mL/min. The inlet temperature for both feed and coolant water was maintained at 20 °C using water baths (model 600-F, Julabo, Germany). Maintaining a fixed inlet temperature was essential to avoid forming a temperature gradient that may lead to offset flux under dark conditions (no illumination).

**Figure 1:**
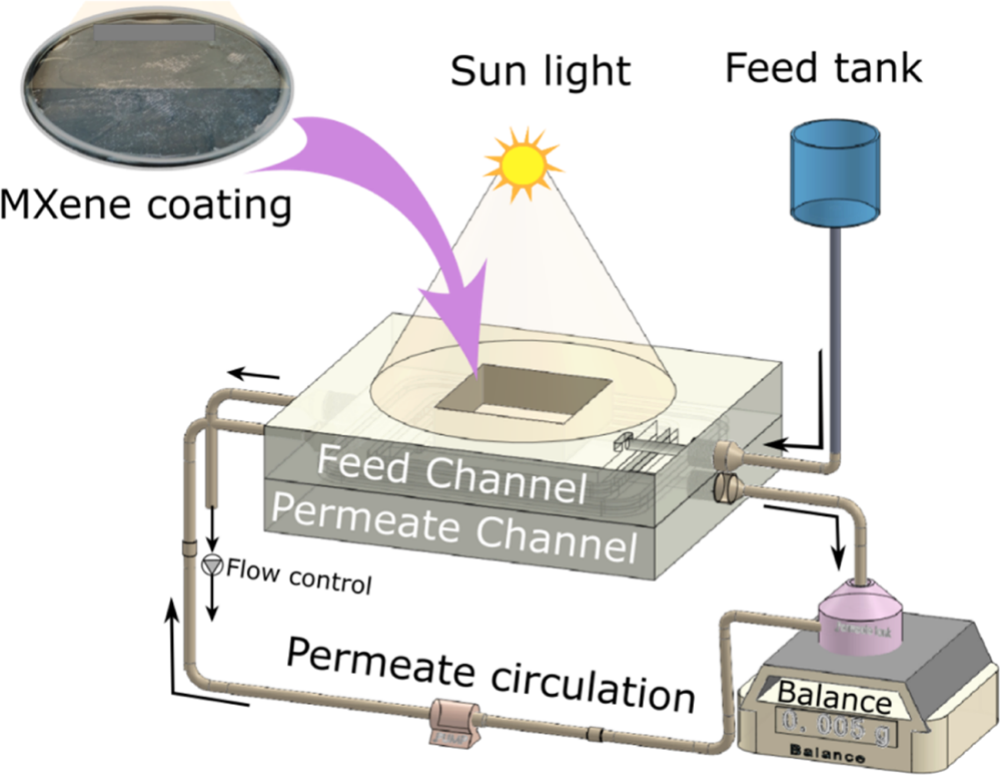
Schematic representation of the MXene-based PMD setup. The feedwater (the top blue tank of 2 L) was supplied to the feed channel using hydrostatic pressure, and the flow was maintained using a needle valve. The permeate was weighed on a computer-interfaced scale. A photograph of the experimental setup can be found in Figure S2B.

During the PMD process, the membranes were illuminated using a solar simulator (OAI, TriSOL, 350W) at an illumination power density of one sun (ca. 1000 W/m^2^). A light meter (Extech-SDL-400) was used to maintain the light intensity at ca. 1000 W/m^2^. A quartz optical window (50 mm × 50 mm) was mounted on the top of the MD module to minimize the absorption of incident light. Figure S1 shows the module geometry and the exposed part for photothermal membrane exposure. A schematic representation of the whole experimental apparatus used to examine the PMD performance of virgin and MXene-coated membranes is depicted in Figures 1 and S2, respectively. To properly evaluate the PMD performance of the fabricated membranes, we simultaneously investigated the impact of the feed concentration and the flow rate. For that, we conducted our studies at four different concentrations (i.e., 0.36, 5, 10, and 20 g/L), and at each of these concentrations, the PMD performance was tested at four different flow rates, that is, 0.1, 2, 4, 8, 12, and 20 mL/min, respectively. The temperatures of the inlet and outlet of feed and coolant streams were recorded using thermistors (NTC, 10K, accuracy = 0.01 °C), and the membrane surface temperature was captured using an infrared (IR) camera (Fluke, TiS40). The distillate mass was weighed using a digital balance logged on a computer (Mettler Toledo, ML6002T, accuracy 0.001 g). The distillate conductivity was monitored using an accurate conductivity meter (WTW 3310) to observe any membrane pore wetting occurrence throughout the MD process.

### 2.5 PMD-Related Calculations

The flux (J, kg/m^2^ h) through the fabricated membranes was estimated using the following equation
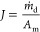
1where *ṁ*_d_ (kg/h) is the distillate flow rate and *A*_m_ (m^2^) is the active membrane surface area exposed to light irradiation. Upon illumination, the MXene-induced photothermal effect provides heat energy to the membrane surface, primarily utilized to increase the feed temperature and, subsequently, the latent heat of evaporation. However, part of this generated heat is typically lost in the conduction heat transfer across the membrane to the coolant side. Thus, the efficiency of freshwater production (energy efficiency, η_th_), which is equivalent to the gain output ratio (GOR) in thermal desalination systems without heat recovery, is defined as the ratio of the heat of distillate water to that of total input energy.^55^ In the case of PMD, the input is light energy; and therefore, the GOR can be expressed as
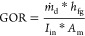
2where *ṁ*_d_ is the rate of distillate water production (kg/s), *h*_fg_ is the enthalpy of vaporization (kJ/kg), and *I*_in_ is the power density of incident light (kW/m^2^). The salt rejection ratio (RR) can be determined using the following formula
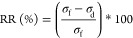
3where σ_d_ and σ_f_  are the distillate and feedwater conductivities (mS/cm), respectively.

## 3 Results and Discussion

### 3.1 MXene Synthesis and Characterization

**Figure 2:**
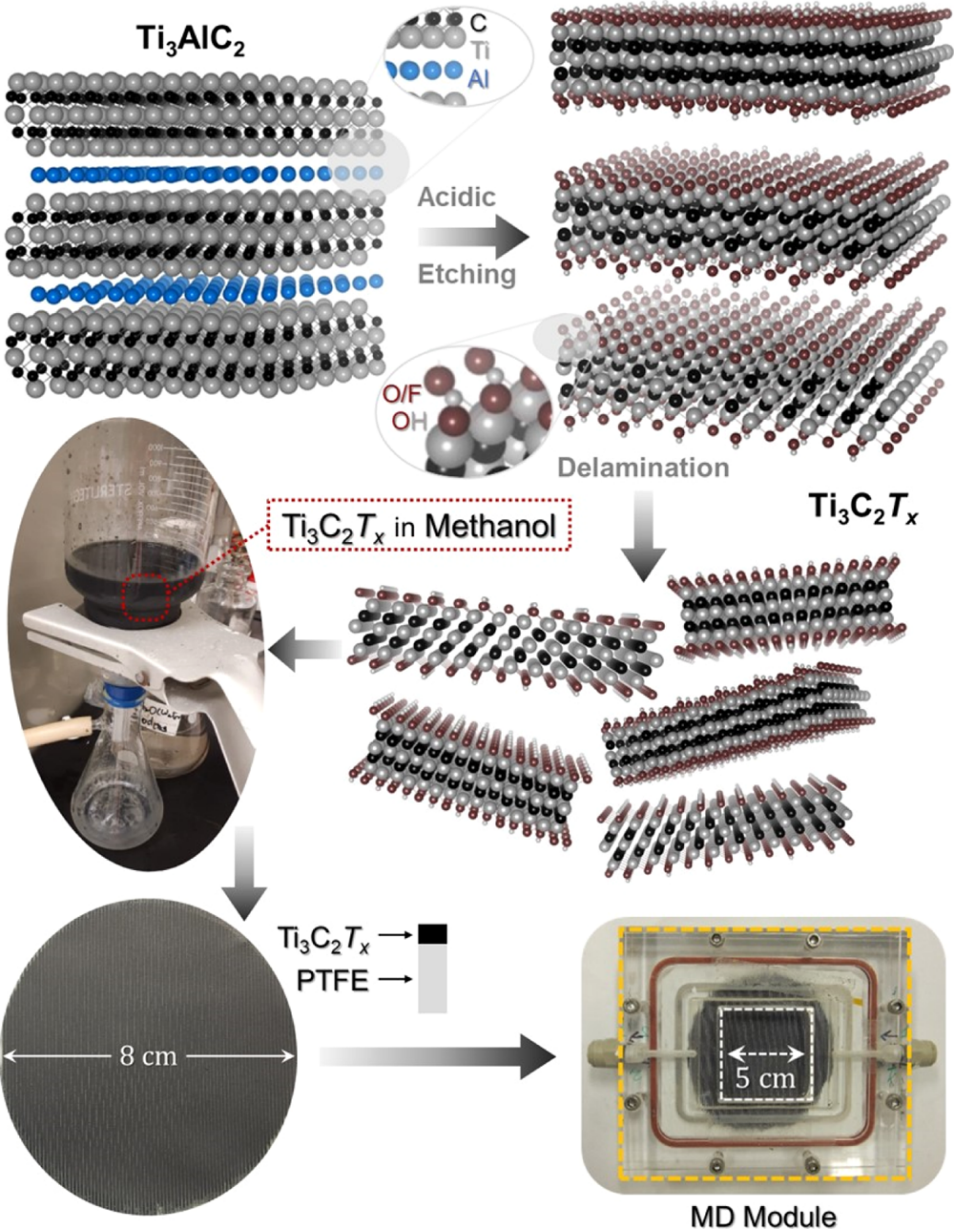
Schematic illustration of the synthesis procedure of freestanding Ti_3_C_2_*T*_*x*_ MXene nanosheets followed by vacuum-assisted filtration to deposit Ti_3_C_2_*T*_*x*_ films on hydrophobic PTFE membranes. The MXene-coated membrane was placed inside the MD module (the yellow dashed boundary in the last panel) using silicone O-rings. The white dashed boundary denotes the effective illumination area (5 cm^2^).

The Ti_3_C_2_*T*_*x*_ MXene nanosheets used in this study were exfoliated by selectively removing the Al layer from their parent MAX phase (i.e., Ti_3_AlC_2_) using chloride- and fluoride-containing etchants. Afterward, as described in the Materials and Methods Section and schematically illustrated in Figure 2, freestanding Ti_3_C_2_*T*_*x*_ nanosheets were obtained using a post-etching Li^+^ ion intercalation process.

**Figure 3:**
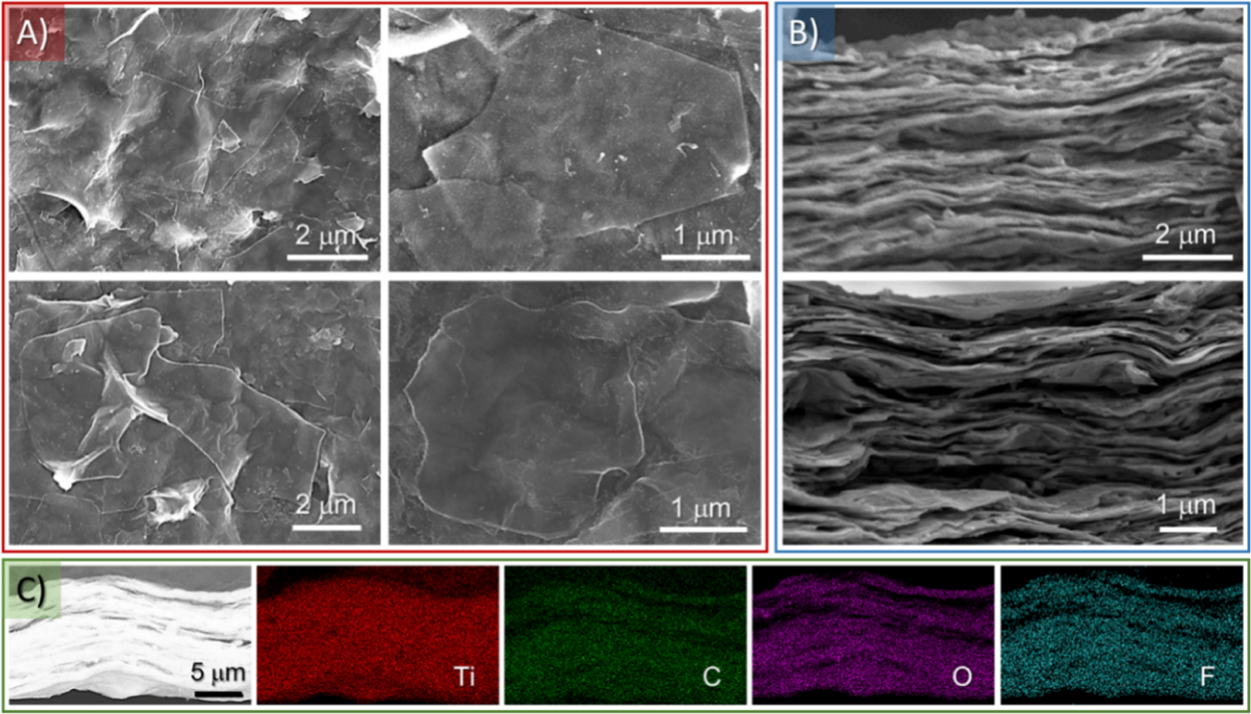
(A) Top-view and (B) cross-sectional SEM micrographs of the MXene coating, taken at different positions. (C) Secondary electron (SE) cross-sectional SEM image along with the corresponding EDX mapping of the characteristic elements of Ti_3_C_2_*T*_*x*_ (i.e., Ti, C, F, and O).

**Figure 4:**
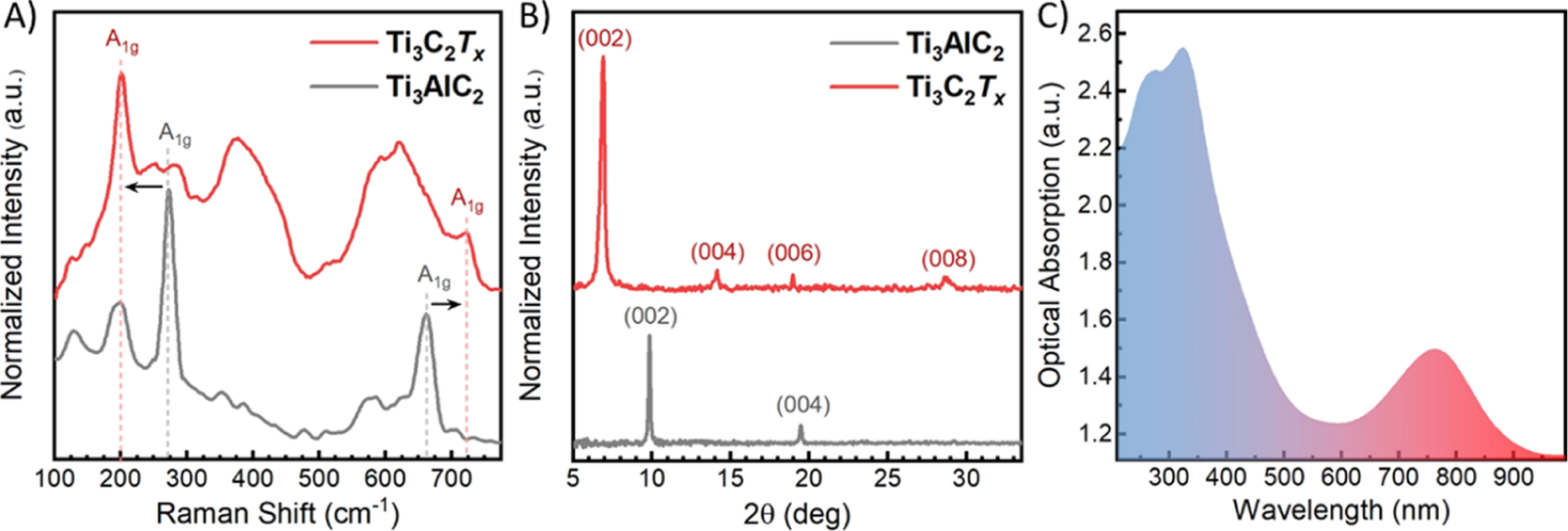
(A) Raman spectra of Ti_3_C_2_*T*_*x*_ MXene (excited at 633 nm) and Ti_3_AlC_2_ MAX phases (excited at 532 nm). (B) XRD patterns of Ti_3_C_2_*T*_*x*_ and Ti_3_AlC_2_ phases. C) UV–vis absorption spectrum of Ti_3_C_2_*T*_*x*_ with its two characteristic bands at ca. 330 and 760 nm, respectively.

Following the vacuum-assisted filtration of our MXene suspensions (in methanol), we investigated the conformality of the Ti_3_C_2_*T*_*x*_ nanosheets over the PTFE membrane using SEM. Figures 3A and S3 present the corresponding top-view SEM micrographs of the deposited nanosheets, respectively, showing their relatively large lateral dimension (ca. 2.5+ μm) and homogeneity over the surface. Despite the hydrophobicity of the PTFE, the Ti_3_C_2_*T*_*x*_ nanosheets have demonstrated excellent preferential alignment along the surface, as shown in the cross-sectional SEM micrograph in Figure 3B. In principle, the attained 2D layered structure can form a network of interchannel passages through which water will easily flow. The stoichiometric uniformity across the cross section of the coated MXene films was confirmed using EDX spectroscopy in conjunction with SEM, as shown in Figure 3C. The corresponding EDX maps (Figure 3C) demonstrate the homogeneous distribution of the characteristic elements constituting the MXene nanosheets, that is, Ti, C, F, and O. The molecular vibrations associated with the bonding between those atoms were also probed utilizing Raman spectroscopy. Figure 4A displays the corresponding Raman spectra of the MAX phase and the well-exfoliated Ti_3_C_2_*T*_*x*_, showing their typical in-plane and out-of-plane Raman-active modes, that is, E_1g_ and A_1g_, respectively, in the range of 100–800 cm^–1^. The observed sharp peaks in the Raman spectrum of the parent Ti_3_AlC_2_ phase at ca. 126, 198, 272, and 662 cm^–1^ are representative of its characteristic molecular vibrations.^56^ As a result of the proper removal of the Al, the A_1g_ (Ti, C, *T*_*x*_: O) and A_1g_ (C) vibrational modes of the MXene at ca. 202 and 720 cm^–1^ are, respectively, stiffened and softened relative to those of the MAX at ca. 272 [A_1g_ (Ti, Al)) and 662 cm^–1^ (A_1g_ (C)]. The other 230–475 cm^–1^ region peaks mark the typical E_1g_ vibrational modes associated with the functional surface species (*T*_*x*_).^57,58^ The remaining peaks in the ca. 530–750 cm^–1^ range are solely related to carbon vibrations.^57^ The obtained active vibrational modes characteristic of Ti_3_C_2_*T*_*x*_ nanosheets correlate well with previous reports.^57−59^

To further verify the openness of the MXene-layered structure, illustrated in Figure 3B, we performed XRD spectroscopy on the synthesized Ti_3_C_2_*T*_*x*_ nanosheets and their parent MAX phase. The corresponding XRD patterns are demonstrated in Figure 4B, showing all the typical diffraction peaks of Ti_3_C_2_*T*_*x*_ in the 5–35° range. Manifestly, the characteristic (002) peak of Ti_3_AlC_2_ was shifted from 2θ = 9.8 to 6.9° for Ti_3_C_2_*T*_*x*_, indicating an increase in the interlayer spacing. The broadening and shift in the characteristic (002) peak are attributed to the substitution of the Al layers with the surface-terminating groups (*T*_*x*_), resulting from the proper exfoliation followed by the delamination. Furthermore, to demonstrate the optical activity of our MXene nanosheets within the solar spectrum, we probed the UV–vis spectral absorption (200–1000 nm) of our Ti_3_C_2_*T*_*x*_ nanosheets. Figure 4c illustrates the broad optical absorption garnered by Ti_3_C_2_*T*_*x*_ MXene with its two characteristic interband transition and out-of-plane plasmonic bands at ca. 330 and 760 nm, respectively.^47,58,60,61^ It is important to remark that the potential photothermal behavior of Ti_3_C_2_*T*_*x*_ is highly affected by these two broad absorption bands, especially the latter as a result of the resonant excitation of the SP oscillations at the 2D surface of the MXene nanosheets.^49,62−64^

### 3.2 Evaluation of Membrane Performance in Desalination

**Figure 5:**
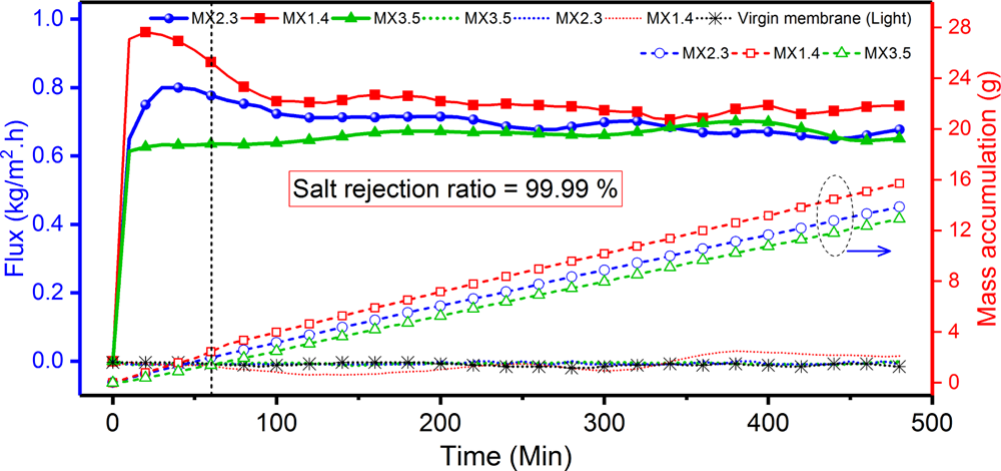
Water vapor flux (left axis) vs PMD operation time and the corresponding mass of accumulated distillate water (right axis) for the three MXene-coated PTFE membranes (under one sun). Note: Almost no flux was attained under dark conditions (dotted lines). At a feed salinity of 0.36 g/L, the recorded ratio of salt rejection was always above 99.99% for all membranes.

The PMD behavior of MXene-coated membranes (MX1.4, MX2.3, and MX3.5) was tested for DCMD desalination under a 1000 W/m^2^ light source (one sun). Figure 5 represents the vapor flux (in the dark and under illumination) and water production (under illumination) generated over an 8 h interval using the three fabricated MXene membranes. For both virgin and MXene-coated membranes, the salt RR calculated from the conductivity data was always above 99.99%. Patently, shining the MXene-coated PTFE membranes with light has led to an omnipresent enhancement in the achieved flux due to the intrinsic photothermal activity of Ti_3_C_2_*T*_*x*_. During the PMD process, water vapor permeated from the feed side through the membrane dry pores under the photothermal-induced temperature gradient. However, interestingly, we found that the flux output is inversely proportional to the MXene aerial densities (mg/cm^2^); the thicker the coating, the higher the mass transfer resistance. That is why the MX1.4 membranes have yielded the highest vapor flux (0.77 kg/m^2^ h), followed by MX2.3 and MX3.5. Once the solar light irradiated the MX1.4 membrane, the flux sharply increased to a maximum value of 0.96 kg/m^2^ h before it slightly decreased and plateaued at an average flux of 0.77 kg/m^2^ h. This slight surge in the flux before the plateau is attributed to the initial low conduction heat at the relatively cold surface. As the surface temperature increases, the conduction heat loss also increases.

For comparison, control experiments were performed using virgin and MXene-coated PTFE membranes under dark conditions, with both exhibiting nearly zero flux (Figure S4). It is worth mentioning that, under one-sun irradiation, the MXene-based system can generate 6 L/day of pure water per one square meter of membrane area (8 h average daytime). This amount is sufficient to meet the drinking water needs of a household.

**Figure 6:**
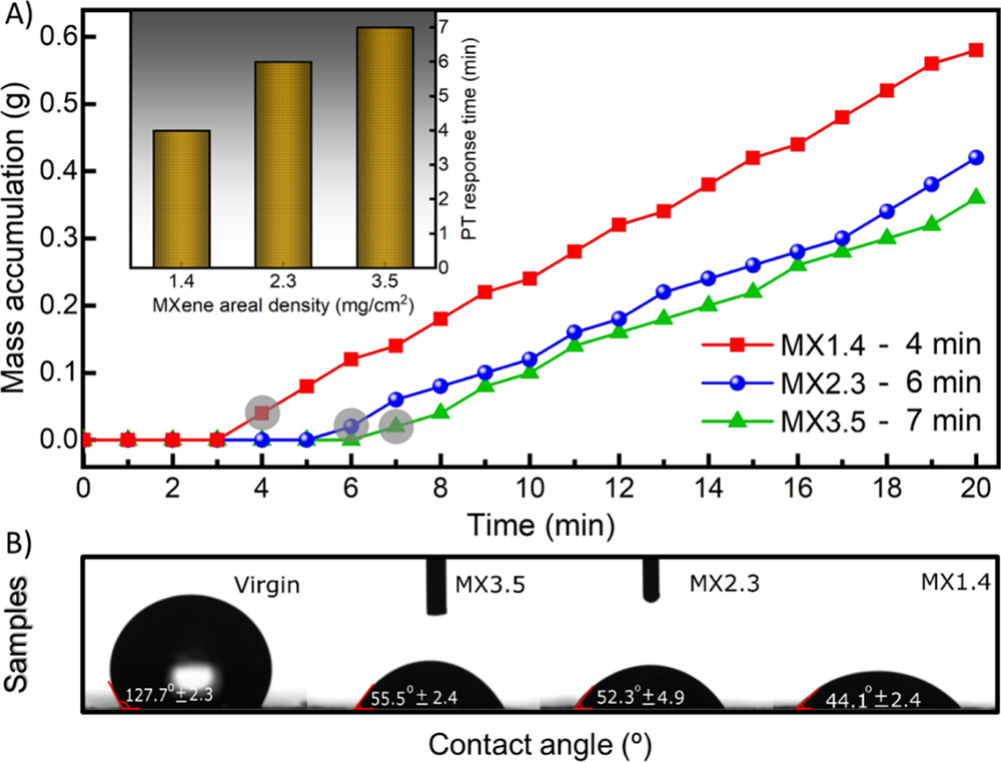
(A) Permeate mass accumulation for the three MXene-coated membranes during the first 20 min of the PMD process. The shaded circles denote the response time taken to produce the first flux. Inset: The dependence of the photothermal response time of the MXene-coated membranes on the areal densities of the Ti_3_C_2_*T*_*x*_ coating (i.e., 1.4, 2.4, and 3.5 mg/cm^2^). (B) Contact angle of the virgin and the three MXene-coated PTFE membranes.

Figure 6A shows that the thicker membrane (MX3.5) exhibited a slower vapor transport which started to produce flux after 7 min, mainly due to the increase in mass transfer resistance. On the other hand, it only took 4 min for the thinner membrane (MX1.4) to generate the first vapor flux, owing to the short vapor path length. As a result, the permeate mass accumulated by the MX1.4 membrane over 20 min was much higher than in the case of its counterparts, thanks to the decreased mass transfer resistance (Figure 6A). Remarkably, the rejection of the MXene-coated membranes did not differ much from the virgin membranes, even though there was a significant decrease in the water contact angle due to the hydrophilic nature of the MXene coating (Figure 6B). The relatively stable permeability indicates that the Ti_3_C_2_*T*_*x*_ coating was mainly formed at the top surface of the PTFE membrane, with minimal penetration into the porous membrane structure, which remained hydrophobic. Hence, the modified membranes maintained their separation function, acting as a barrier to liquid water passage while providing self-heating behavior. Because of its higher performance, the MX1.4 membrane was used in the subsequent experiments.

### 3.3 Photothermal Response under Intermittent Lighting

**Figure 7:**
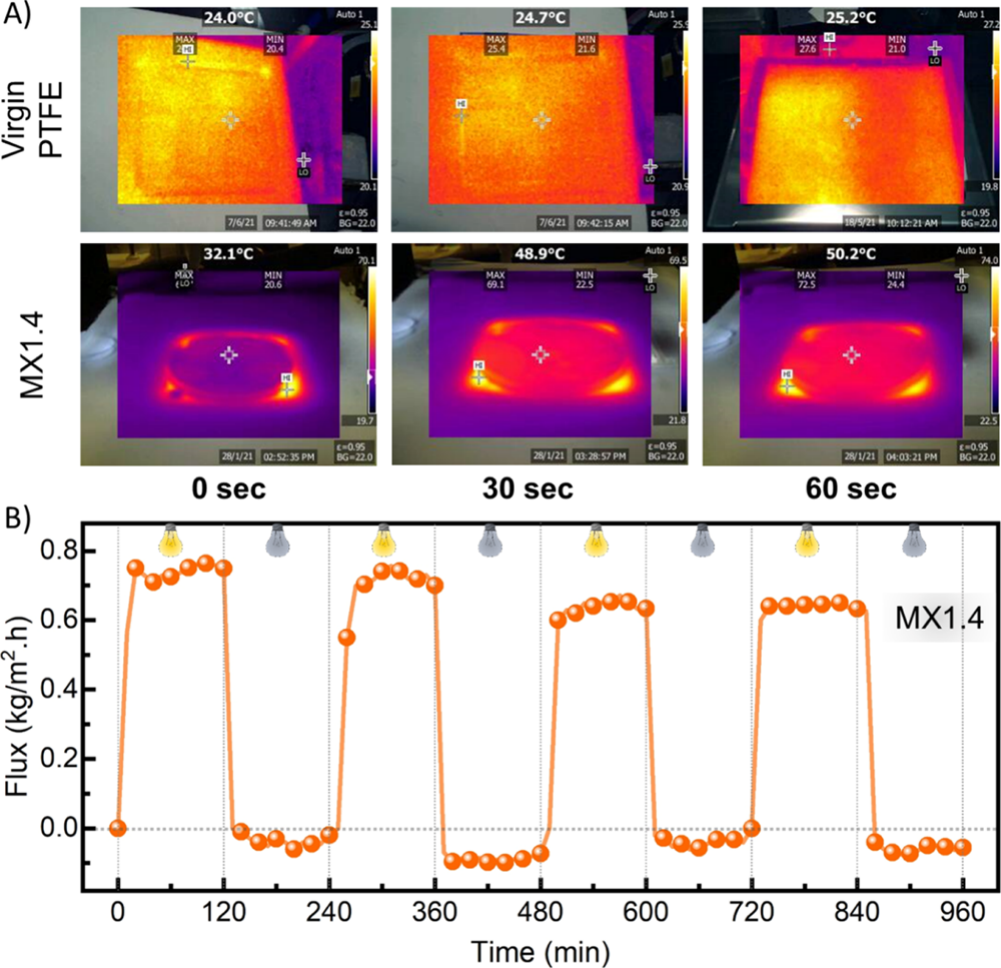
(A) IR images of the photothermal time response of the virgin PTFE (top) and MX1.4 (bottom) membranes during the PMD process upon one-sun illumination. Within 60 s, the temperature of the MXene-coated membrane (MX1.4) reached 50.2 °C, while the virgin membrane remained almost unchanged at 25.2 °C. (B) Photothermally induced permeate flux of the MX1.4 membrane under intermittent lighting, at a feed salinity of 0.36 g/L.

To highlight the intrinsic fast photothermal response of the Ti_3_C_2_*T*_*x*_-coated membranes compared to virgin ones, we used a thermal camera to record the IR images of both membranes during the PMD process (Figure 7A). Notably, the MX1.4 membrane has demonstrated an impressive fast light-to-heat conversion, where the temperature jumped to 50.2 °C within 60 s once irradiated with a one-sun light source. Meanwhile, the temperature of the virgin membrane has barely increased by 1 °C. To mimic the performance of the PMD system under typical daytime and night-time conditions, we examined the response of the MX1.4 membranes under intermittent lighting (ON/OFF) every 2 h, as displayed in Figure 7B. In a typical experiment, once the light was ON, the inherent photothermal activity of the PMD membrane stimulated the heat transfer through the water meniscus (a water film thickness of 4.5 mm) formed at the MXene-coated membrane interface and promoted the evaporation process across the porous membrane. After 2 h, the light was switched off, leading to a sharp decrease in the flux to almost zero due to the absence of external heat supply. The experiment was repeated four times under alternating ON and OFF light conditions, yielding an average water vapor flux of ca. 0.67 kg/m^2^ h, obtained over the four cycles. Markedly, when we applied different ON/OFF cycles, the flux response to the lighting conditions was almost instantaneous and identical. The observed trend correlates with previous theoretical and experimental reports on the dynamic time response of MD systems under bulk heating.^6500–6502^

### 3.4 Effect of Feed Flow Rate and Salinity

**Figure 8:**
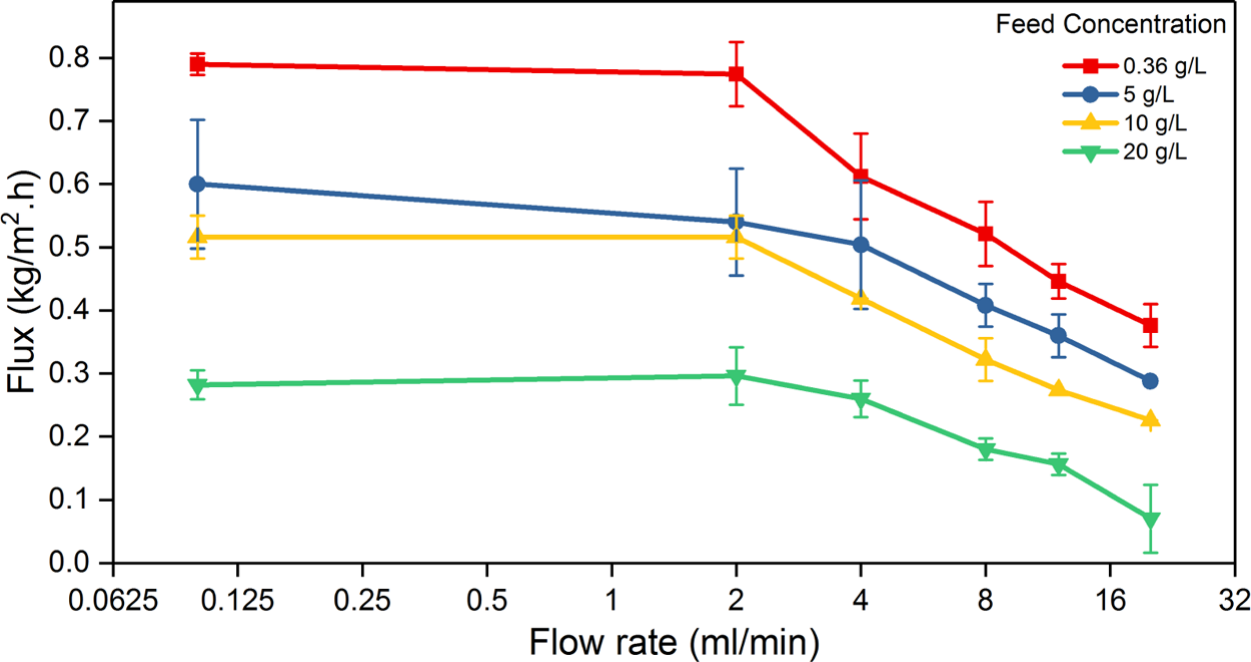
Water vapor flux versus feed flow rates for different feedwater concentrations under one-sun irradiation. As the feed concentration increases, the vapor flux decreases. The decrease in flux was sharp for the feed flow rate above 2 mL/min.

In conventional MD systems, higher feed crossflow velocities yield higher flux due to enhanced feed mixing and heat transfer, leading to lower TP and lower residence time.^18,65^ However, in the presence of localized heating, as in PMD systems, this trend is reversed; the flux is directly proportional to the residence time. In principle, given that the membrane is the heating source, a longer contact time of the feedwater is needed to induce sufficient heat gain. In other words, a longer residence time at the membrane interface is imperative for enhanced water vapor flux.^65,66^ Following the same norm, MXene-coated PMD membranes subjected to slower feed flow rates (i.e., from 0.1 to 2 mL/min) have produced higher and steady distillate flux, as shown in Figure 8. In contrast, the generated flux has expressed a monotone decrease (a slope of ca. 0.45) for flow rates higher than 2 mL/min (up to 20 mL/min). This decrease in the flux with the same trend for different feed concentrations is expected to continue at higher flow rates. By increasing the feed flow rate, the contact time of feedwater with the PMD membrane surface decreases. It is worth noting that there was an increase in the flux initially due to changing the flow rate from zero to 0.1 mL/min. This increase was ascribed to the reduction in the concentration polarization, which is usually higher when feedwater is stagnant.

Also, as shown in Figure 8, the feed concentration has significantly influenced the membrane performance. At any flow rate, the overall distillate flux was inversely proportional with the salinity of the feed solution (i.e., 0.36, 5, 10, and 20 g/L). However, for a given NaCl concentration, the impact of the flow rate remained unchanged. Thus, at feed flow rates in the range of 0.1–2 mL/min and the feed concentration of 0.36 g/L, the Ti_3_C_2_*T*_*x*_-coated PMD membrane exhibited the highest flux value of ca. 0.77 kg/m^2^ h. The flux has then dropped to 0.58, 0.52, and 0.28 kg/m^2^ h at a feed salinity of 5, 10, and 20 g/L, respectively (Figure 8)*.* Such salinity-dependent behavior accords with the following: (1) the increase in the salt content in the feed solution would generally decrease the rate of water evaporation. (2) The accumulation of the salt particles during the PMD process (Figure S5) enhances the concentration polarization effect and reduces the light exposure to the MXene coating. (3) The accumulated salt would partially block the irradiated light and produce more scattering, especially at the membrane/water interface, which would minimize the MXene-induced photothermal effect, that is, the driving force of the PMD process, hence the distillate flux. This phenomenon is confirmed by the observed temperature decrease at the membrane surface when feed concentration increases.

### 3.5 Energy and Photothermal Efficiency

Theoretically, the total preserved energy in the coolant fluid is the sum of the latent heat of condensation and the heat of conduction from the feed side. Thus, the total heat energy transfer from the feed side can be expressed as follows
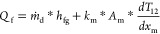
4

**Figure 9:**
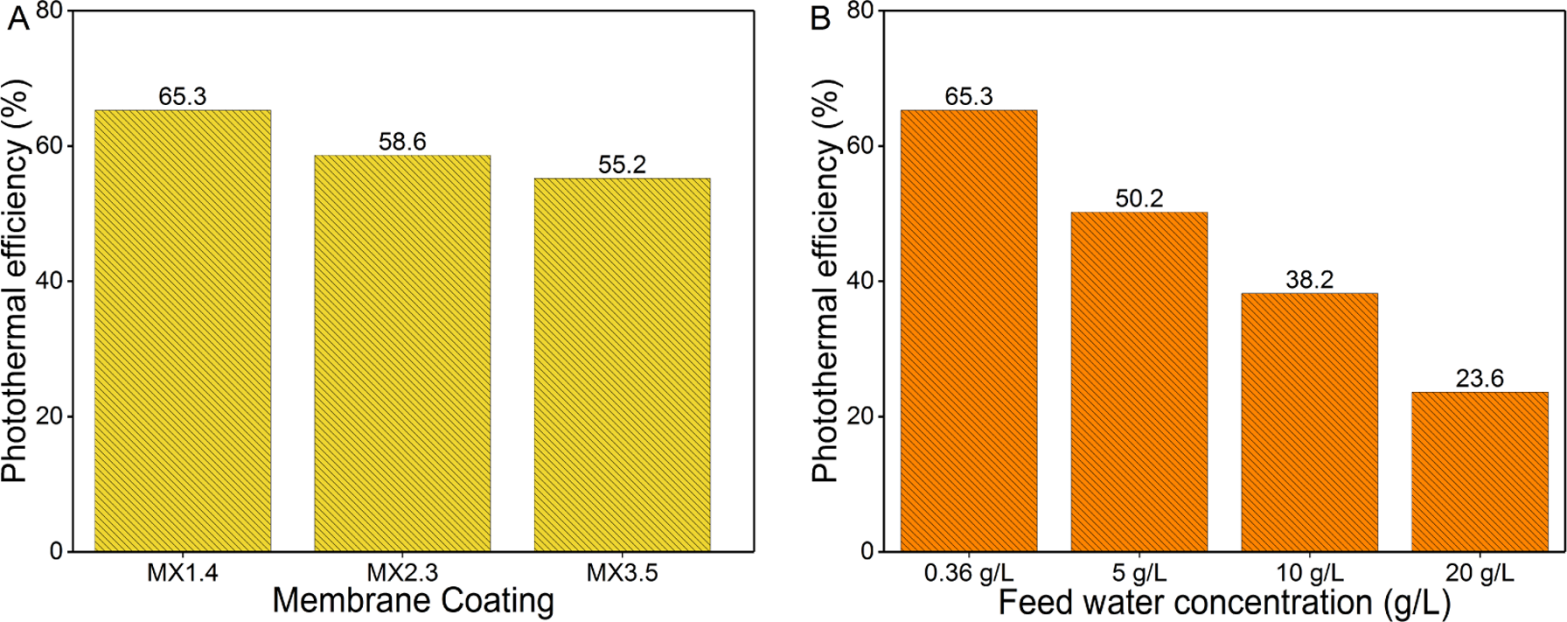
(A) Photothermal efficiency of different MXene coatings showing a decrease as aerial MXene density increases. (B) Photothermal efficiency of MX1.4 coating with different feed concentrations. The photothermal efficiency decreases as the feed concentration increases.

At the permeate side, the heat energy gain can be expressed as
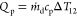
5where Δ*T*_12_ is the temperature difference (°C) between the inlet and outlet of the coolant stream, *c*_p_ is the specific heat of water (kJ/kg °C), *k*_m_ is the coefficient of heat transfer across the membrane (W/mK), and *x*_m_ is the thickness of the membrane (m). On the other hand, the photothermal efficiency of the membranes can be deduced from the ratio of the energy of distillate water to the total incident light absorbed by the MXene-coated PMD membrane, as follows
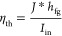
6where *h*_fg_ is the enthalpy of vaporization (kJ/kg) and *I*_in_ is the power density of incident light (kW/m^2^). Figure 9A presents the photothermal efficiencies of different MXene-based membranes at a feed salinity of 0.36 g/L. As expected, the MX1.4 membrane has exhibited the highest efficiency of ca. 65.3% among the MXene-coated membranes. Analogous to the flux data in Figure 8, the calculated photothermal efficiency of MX1.4 has also declined by ca. 64% upon increasing the salinity of the feedwater to 20 g/L, as shown in Figure 9B. The corresponding photothermal efficiency obtained at each salinity is mentioned in Figure 9B. The reduction in the photothermal efficiency agrees with our previous explanation regarding the blocked/scattered irradiated light by salt molecules.

For comparison, it is essential to note that although the present study shows a photothermal efficiency similar to the only reported PMD using MXene,^26^ the incident light input used in our work was significantly lower (ca. 6 times less). Their flux was much higher, although because of their preheated inlet feedwater (at 65 °C) and the higher light power density (5.8 suns) at which their MXene-coated membranes (PVDF in their case) were irradiated. However, for other non-MXene-based MD systems irradiated at lower power densities (i.e., 0.75–one sun) with a feed temperature of ca. 20 °C, the reported water vapor fluxes and photothermal efficiencies were on par with our results. Given the nascent stage of research on MXene-based MD systems, meeting the performance of other mature photothermal materials is quite recognizable. Nonetheless, whether further research should be directed to developing novel photothermal materials with ideal intrinsic properties to effectively enhance PMD performance or invest in designing innovative modules and optimized processes toward high-performance PMD systems remains a debatable question.^67^

## 4 Conclusions

In this work, we prepared three sets of MXene-coated membranes, each with a specific aerial density, where the hydrophobic PTFE membranes were successfully coated with the hydrophilic Ti_3_C_2_*T*_*x*_ MXene at different aerial densities using an optimized vacuum-assisted filtration technique. We investigated their PMD performance in a DCMD system under one-sun illumination. Membranes prepared with the least MXene loading (1.4 mg/cm^2^) have yielded the highest water vapor flux of ca. 0.77 kg/m^2^ h and the best photothermal efficiency of ca. 65.3% at a feed concentration of ca. 0.36 g/L. Our findings showed that feed flow rates above 2 mL/min have negatively affected the flux, contradicting conventional MD systems. We have also illustrated how high feed salinity, for example, 10 g/L, has affected the vapor flux of our membranes, bringing it down by ca. 40%, with a photothermal efficiency of ca. 38%. Similarly, an increase in the salinity of the feedwater to 20 g/L has significantly reduced the flux by ca. 63.8%. This reduction was attributed to the decreased temperature of the water meniscus at the membrane interface caused by the scattering of incident light by the accumulated salt particles on the top of the MXene nanosheets. In addition, the flux produced by our self-heating PMD system has demonstrated an instantaneous response to intermittent illumination, highlighting the capability of our system to operate autonomously in off-grid remote areas. Interestingly, our proposed solar-driven PMD system can produce ca. 6 L/day of freshwater per meter square of membrane surface area without additional external heating. Ultimately, we believe that there is further room to maximize the performance of such a cost-efficient PMD system by optimizing the optical absorptivity of our MXene nanosheets, thanks to their widely tunable properties.

## Data Availability

The data sets generated and/or analyzed during the current study are available upon reasonable request.

## Supporting Information Available

Sketches and photographs of the used experimental setups and additional material characterization for the MXene-coated membrane and its performance (PDF)
