# Supplementary material for: MXene-Coated Membranes for Autonomous Solar-Driven Desalination
Source: ACS Appl Mater Interfaces. 2022 Jan 21;14(4):5265–74. doi: 10.1021/acsami.1c20653 (PMC8815036; doi:10.1021/acsami.1c20653)
Supplement: Supplementary file 1 — am1c20653_si_001.pdf [file am1c20653_si_001.pdf]

Supplementary Information

MXene-coated Membranes for Autonomous Solar-driven Desalination

Mustakeem Mustakeem[1], Jehad K. El-Demellawi[2], M. Obaid[1], Fangwang Ming[2], Husam N. Alshareef[2*], Noreddine Ghaffour[1*]

[1] King Abdullah University of Science and Technology, (KAUST), Water Desalination and Reuse Center (WDRC), Biological and Environmental Science and Engineering Division (BESE), Thuwal 23955-6900, Saudi Arabia

[2] King Abdullah University of Science and Technology, (KAUST), Physical Sciences and Engineering (PSE) Division, Thuwal 23955-6900, Saudi Arabia

**E-mails: husam.alshareef@kaust.edu.sa; noreddine.ghaffour@kaust.edu.sa*

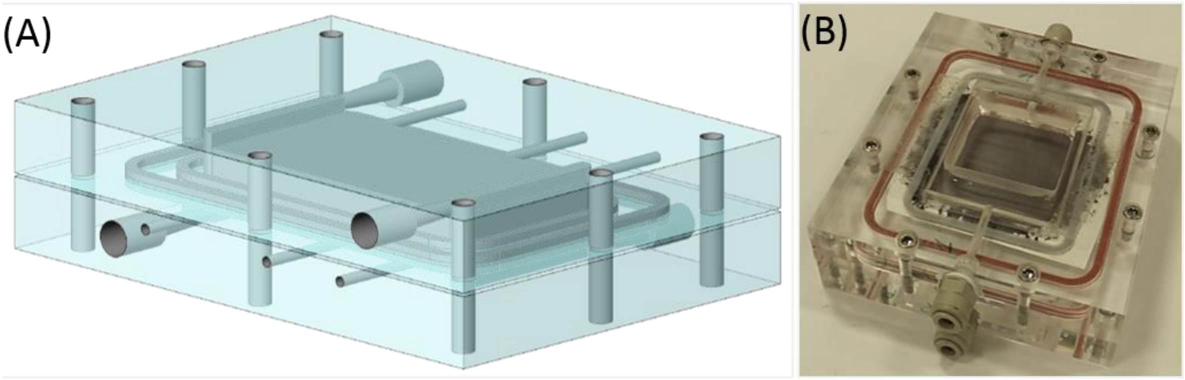

Fig. S1. A) A SolidWorks® sketch of the MD module. B) Photograph of the PMD module embedded with the commercial PTFE membrane coated with $Ti_3C_2T_x$ MXene.

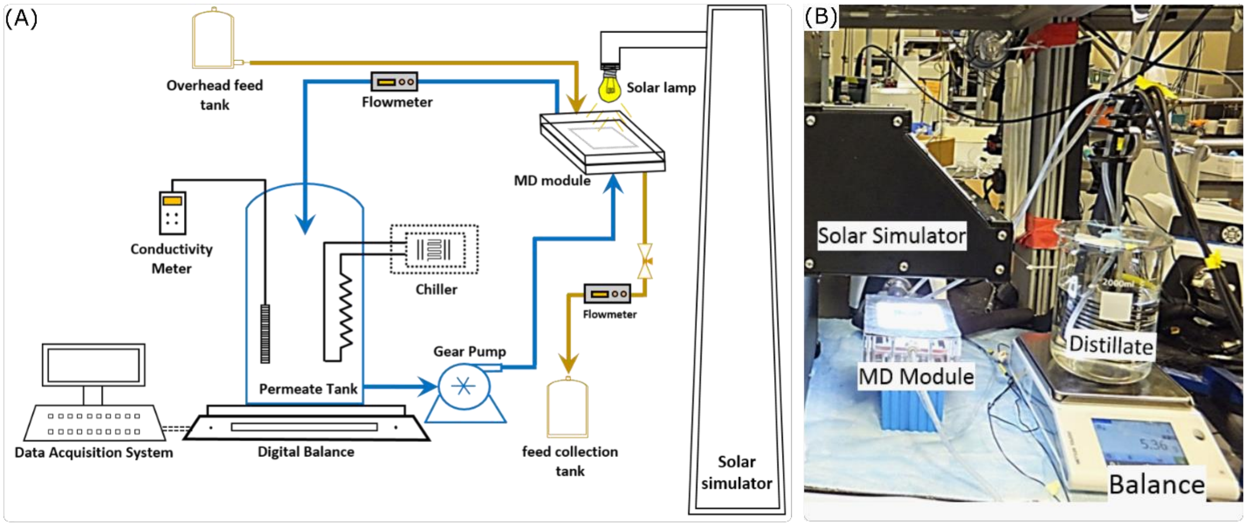

Fig. S2. A) A schematic representation of a typical MD system. B) A photograph of the PMD setup used in this study.

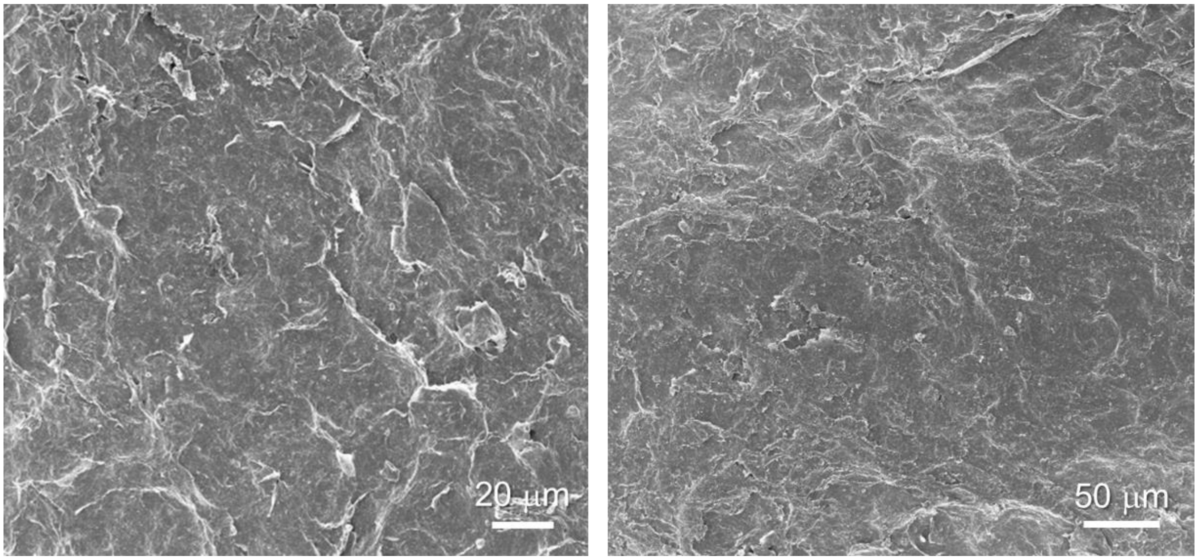

Fig. S3. Low-magnification top-view SEM micrographs of the MXene-coated PTFE membranes, showing the homogeneity of the MXene coating over the surface, with no protruding nanosheets.

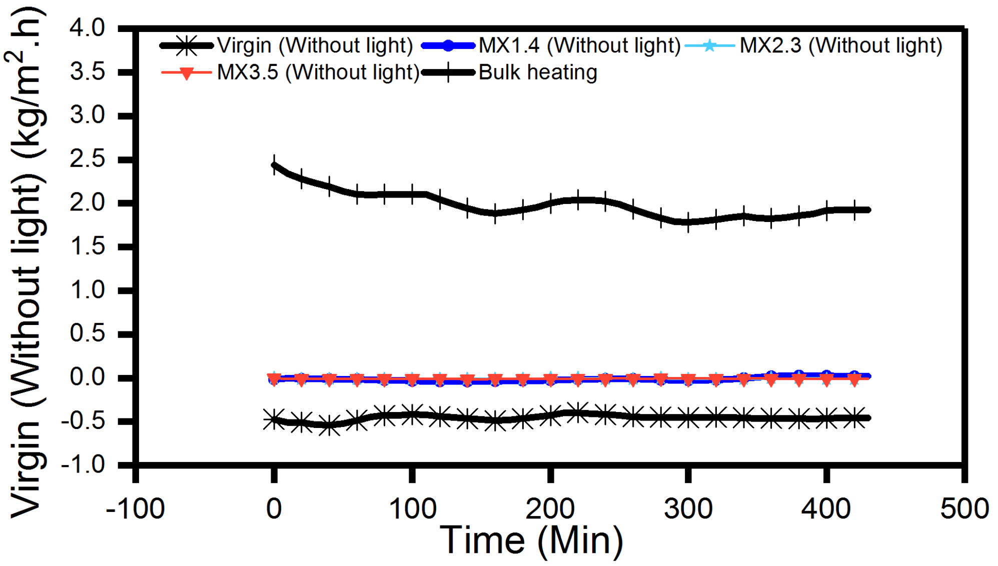

Fig. S4. The dark flux (without illumination) of the MD system using a virgin PTFE membrane in the presence and absence of bulk heating (45 °C feed temperature and 23 °C permeate temperature, at a feed flow rate of 100 mL/min) and self-heating MXene-coated membranes.

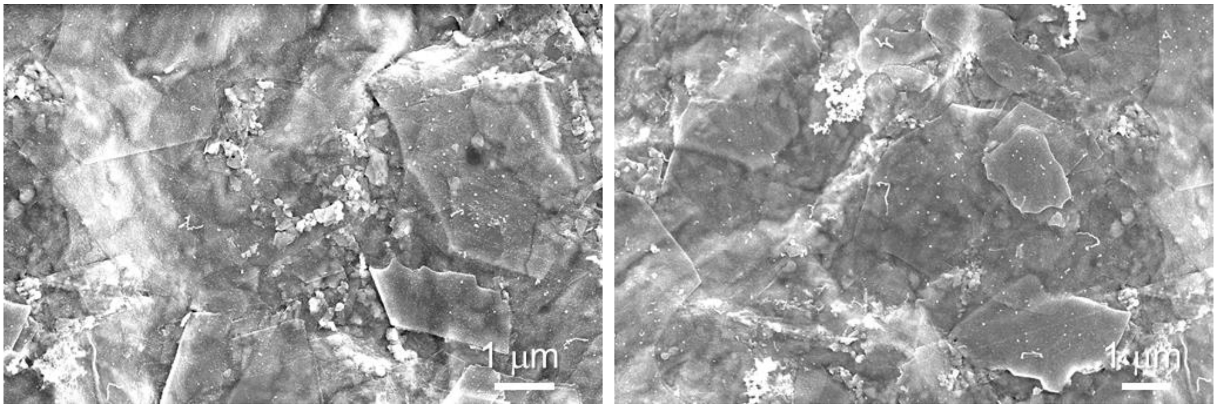

Fig. S5. Top-view SEM micrographs of the MXene-coated PTFE membranes, showing the accumulation of the salt (NaCl) particles on the top of the $Ti_3C_2T_x$ film at the end of the PMD process.
